# Acquisition of resistance to avian leukosis virus subgroup B through mutations on *tvb* cysteine-rich domains in DF-1 chicken fibroblasts

**DOI:** 10.1186/s13567-017-0454-1

**Published:** 2017-09-13

**Authors:** Hong Jo Lee, Kyung Youn Lee, Young Hyun Park, Hee Jung Choi, Yongxiu Yao, Venugopal Nair, Jae Yong Han

**Affiliations:** 1 Department of Agricultural Biotechnology, College of Agriculture and Life Sciences, and Research Institute of Agriculture and Life Sciences, Seoul National University, Seoul, 08826 South Korea; 2 The Pirbright Institute, Woking, Pirbright, Surrey, GU24 0NF UK; 3 Institute for Biomedical Sciences, Shinshu University, Minamiminowa, Nagano, 399-4598 Japan

## Abstract

Avian leukosis virus (ALV) is a retrovirus that causes tumors in avian species, and its vertical and horizontal transmission in poultry flocks results in enormous economic losses. Despite the discovery of specific host receptors, there have been few reports on the modulation of viral susceptibility via genetic modification. We therefore engineered acquired resistance to ALV subgroup B using CRISPR/Cas9-mediated genome editing technology in DF-1 chicken fibroblasts. Using this method, we efficiently modified the tumor virus locus B (*tvb*) gene, encoding the TVB receptor, which is essential for ALV subgroup B entry into host cells. By expanding individual DF-1 clones, we established that artificially generated premature stop codons in the cysteine-rich domain (CRD) of TVB receptor confer resistance to ALV subgroup B. Furthermore, we found that a cysteine residue (C80) of CRD2 plays a crucial role in ALV subgroup B entry. These results suggest that CRISPR/Cas9-mediated genome editing can be used to efficiently modify avian cells and establish novel chicken cell lines with resistance to viral infection.

**Electronic supplementary material:**

The online version of this article (doi:10.1186/s13567-017-0454-1) contains supplementary material, which is available to authorized users.

## Introduction

Avian leukosis virus (ALV) is a retrovirus that infects avian species, eventually causing tumors [1]. The ALV is a group VI virus of the family *Retroviridae*, and it can be divided into six subgroups, A–E and J, based on retroviral envelope glycoproteins that play a crucial role in host–virus interactions [2]. ALV-infected poultry display several symptoms, including lymphoblastic, erythroblastic and osteopetrotic tumors, and the virus can be transmitted both vertically and horizontally. The spread of ALV in poultry flocks therefore causes tremendous economic losses within the poultry industry [3].

Susceptibility and resistance to the virus depend largely on specific host receptors that interact with viral envelope proteins. Naturally occurring genetic mutations in the host receptors, or artificial expression of mutant receptors in host cells, can affect susceptibility to the virus. A four base pair (bp) insertion and 1 bp substitution in the tumor virus locus A (*tva*) gene confer resistance to ALV subgroup A [4], and mutations in the first intron of *tva* are also reported to reduce susceptibility to ALV subgroup A [5, 6]. Chickens with a 1 bp substitution in *tvb* creating an in-frame stop codon exhibit complete resistance to ALV subgroup B, and a single amino acid substitution (C125S) reduces susceptibility to ALV subgroups B, D and E [7, 8]. Resistance to ALV subgroup C is closely related to a 1 bp substitution in tumor virus locus C (*tvc*) that creates an in-frame stop codon [9]. Moreover, comparative studies suggest that variation in tryptophan 38 (W38) in the NHE1 gene explains the differences in susceptibility to ALV subgroup J among avian species [10, 11].

Despite the discovery of specific host receptors that are critical for ALV entry, there has been only one report on the acquisition of resistance to ALV subgroup C in avian species via genome editing of host receptor genes. This can be attributed partly to the lack of an efficient genome editing technology [9]. The recently developed clustered regularly interspaced short palindromic repeats (CRISPR)/CRISPR-associated (Cas9) system is a programmable genome editing technology [12] that has been widely adopted for use in many organisms, including mice, fish, pigs and cows [13–16]. Among avian species, CRISPR/Cas9 has also been used successfully for genome editing in chickens [17, 18].

We performed genome editing on the chicken host receptor gene *tvb*, which is related specifically to ALV subgroup B. Since chickens with premature stop codons in CRDs of TVB receptors exhibit resistance to ALV subgroup B, we sought to identify artificial mutations in CRDs of TVB receptors that cause similar effects [7]. We adopted the CRISPR/Cas9 system, an efficient programmable genome editing tool, for use in DF-1 chicken fibroblasts. We then evaluated the susceptibility of genetically modified hosts to ALV subgroup B using flow cytometry.

## Materials and methods

### Experimental animals and animal care

The care and experimental use of chickens were approved by the Institute of Laboratory Animal Resources, Seoul National University (SNU-150827-1). Chickens were maintained according to a standard management program at the University Animal Farm, Seoul National University, Korea. The procedures for animal management, reproduction and embryo manipulation adhered to the standard operating protocols of our laboratory.

### Construction of CRISPR/Cas9 expression vectors

We constructed all-in-one CRISPR/Cas9 vectors targeting *tvb*, with minor modifications. The CRISPR kit used for constructing multiplex CRISPR/Cas9 vectors was a gift from Takashi Yamamoto (Addgene Kit #1000000054) [19], and a neomycin resistance gene under the regulation of a thymidine kinase promoter was inserted into CRISPR/Cas9 vectors by *NotI* digestion and ligation (New England Biolabs, Ipswich, MA, USA). For the insertion of guide RNA sequences into CRISPR/Cas9 vectors, we synthesized sense and antisense oligonucleotides (Bionics, Seoul, Korea) and carried out annealing using the following thermocycling conditions: 30 s at 95 °C, 2 min at 72 °C, 2 min at 37 °C and 2 min at 25 °C. The oligonucleotides used are listed in Table 1.

**Table 1:** **Primers used in this study**

Primers	Sequence
TVB #1 F	5′- CAC CGG CAG CTG AGC GCA TCG TGC G -3′
TVB #1 R	5′- AAA CCG CAC GAT GCG CTC AGC TGC C -3′
TVB #2 F	5′- CAC CGA ATG ACT TTC CCA AGT GCC T -3′
TVB #2 R	5′- AAA CAG GCA CTT GGG AAA GTC ATT C -3′
TVB #1 seq F	5′- AGC TGT CAG CTG GTG GAG TTC AC -3′
TVB #1 seq R	5′- ATA GCG TCC AAT CTG GGT GAG CC -3′
TVB #2 seq F	5′- TCT CCA CGT CTC GGC AGC AC -3′
TVB #1 seq R	5′- CAG CTC TGC TCG GGC TCT CC -3′

### Culture of DF-1 chicken fibroblasts

DF-1 cells were maintained and subpassaged in Dulbecco’s minimum essential medium (DMEM; Hyclone, Logan, UT, USA), supplemented with 10% fetal bovine serum (FBS; Hyclone) and 1× antibiotic–antimycotic (ABAM; Thermo Fisher–Invitrogen, Carlsbad, CA, USA). DF-1 cells were cultured in an incubator at 37 °C in an atmosphere of 5% CO_2_ at 60–70% relative humidity.

### Culture of White Leghorn (WL) chicken embryonic fibroblasts (CEFs)

All internal organs and limbs were removed from WL chicken embryos of 6-day-incubated fertilized eggs, and the remaining embryonic body was then dissociated using 0.05% (v/v) trypsin/ethylenediaminetetraacetic acid (Gibco, Grand Island, NY, USA) at 37 °C for 15 min. The limbs were used for genomic DNA extraction, and the dissociated cells were filtered through 70 mm nylon mesh filters and cultured in DMEM (Hyclone) containing 10% FBS (Hyclone) and 1% ABAM (Thermo Fisher–Invitrogen) in a 5% CO_2_ atmosphere at 37 °C [20].

### Transfection and G418 selection of DF-1 cells

CRISPR/Cas9 vectors (3 µg) were mixed with Lipofectamine 2000 reagent (Thermo Fisher–Invitrogen) in Opti-MEM (Thermo Fisher–Invitrogen), and the mixture was applied to 5 × 10^5^ DF-1 cells. Then, 6 h after transfection, transfection mixtures were replaced with DF-1 culture medium. Geneticin® Selective Antibiotic (G418; GIBCO Invitrogen, Grand Island, NY, USA) (300 µg/mL) was added to the culture medium 1 day after transfection. The complete selection period required up to 7 days.

### T7E1 assay

We adapted the T7E1 assay method from previous publications with minor modifications [21]. Genomic DNA was extracted from DF-1 cells after G418 selection. Genomic regions encompassing the CRISPR/Cas9 target sites were amplified using specific primer sets (Table 1). The amplicons were reannealed to form a heteroduplex DNA structure after denaturation. Subsequently, the heteroduplex amplicons were treated with 5 units T7E1 endonuclease (New England Biolabs) for 20 min at 37 °C and then analyzed by 1% agarose gel electrophoresis.

### Culture of single DF-1 cells and genomic DNA sequencing

After G418 selection, single DF-1 cells from the DF-1 cells treated with CRISPR vectors were seeded in individual wells of a 96-well plate with 100 µL culture medium. We checked the wells each day after seeding and, when the cells in each well were confluent, subpassaged the cells into a 48-well plate. These cells were then used for genomic DNA extraction. The genomic regions encompassing the CRISPR/Cas9 target sites in DF-1 and WL CEFs were amplified using specific primer sets (Table 1), and the PCR products were sequenced using the ABI Prism 3730 XL DNA Analyzer (Thermo Fisher–Applied Biosystems, Foster City, CA, USA). The sequences were analyzed against assembled genomes using BLAST (http://blast.ncbi.nlm.nih.gov).

### Virus production and infection

RCASBP-(B)-CN-EGFP was kindly provided by Dr. Yao and Dr. Nair (Pirbright Institute). CRISPR/Cas9 vectors (5 µg) were mixed with Lipofectamine 2000 reagent (Thermo Fisher–Invitrogen) in Opti-Mem (Thermo Fisher–Invitrogen), and the mixture was applied to 1 × 10^6^ DF-1 cells. The mixture was replaced with DF-1 culture medium 6 h after transfection. One day after transfection we could detect green fluorescence in DF-1 cells, which indicated virus production. Cells were subpassaged, and the medium was changed 1 day after subpassaging. One day later, the medium containing virus was harvested and frozen at −70 °C until use. For viral infection, the medium containing virus was thawed at 37 °C and added to individual DF-1 and WL CEF clones. Four days post-infection, DF-1 and WL CEFs were observed using fluorescence microscopy (TU-80; Nikon, Tokyo, Japan) and analyzed using FACSCalibur (BD Biosciences, San Jose, CA, USA).

### Protein alignment and structure analysis

The protein sequences and bisulfide bond structures of human DR5 TRAIL receptor (NP_003833.4), mouse Tnfrsf10b (NP_064671.2), western clawed frog tnfrsf10b (NP_001004894.1), chicken TVB^S1^ (NP_989446.2) and chicken TVB^S3^ were analyzed using ClustalW.

### Statistical analysis

Statistical analysis system (SAS) software was used for analysis of ALV subgroup B susceptibility. Each treatment was compared using the least-squares method or Duncan’s method, and the significance of the main effects was determined using analysis of variance in the SAS package. A *p* value < 0.05 was regarded as statistically significant.

## Results

### Virus production in DF-1 cells

**Figure 1:**
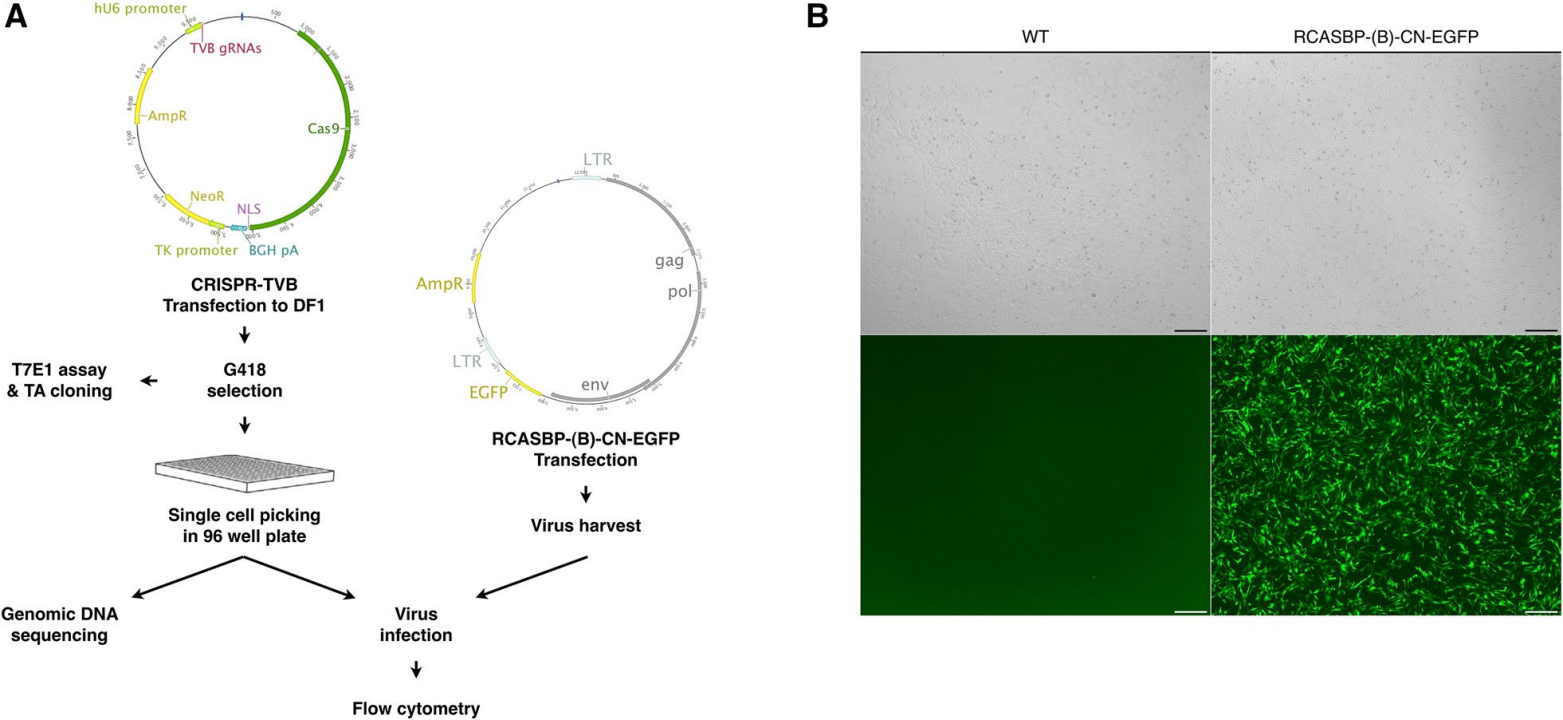
**Schematic representation of this study and virus production in DF-1 cells by RCAS vectors. A** Overview of this study. The CRISPR/Cas9 vectors including Cas9 protein-coding sequences, *tvb*-targeting guide RNA and neomycin resistance genes were transfected into DF-1 cells. After G418 selection, T7E1 assays and TA cloning were performed. *tvb*-modified single DF-1 cells were cultured in 96-well plates, and *tvb* from individual DF-1 clones was sequenced. Clones were infected with ALV subgroup B produced by RCASBP-(B)-CN-EGFP vector-transfected DF-1 cells. **B** ALV subgroup B production in DF-1 cells. DF-1 cells transfected with RCASBP-(B)-CN-EGFP vectors expressed green fluorescent protein (GFP). Non-transfected DF-1 cells (WT) used as negative control. Scale bar = 200 µm.

To produce ALV subgroup B in chicken DF-1 cells, cells were transfected with the RCASBP-(B)-CN-EGFP vector. This vector contains a green fluorescent protein (GFP)-expressing cassette with ALV subgroup B *gag*, *env* and *pol* genes (Figure 1A). This allowed us to assess virus production in DF-1 cells based on GFP expression compared with wild type (WT) DF-1 cells (Figure 1B).

### Genome editing in tvb mediated by CRISPR/Cas9

**Figure 2:**
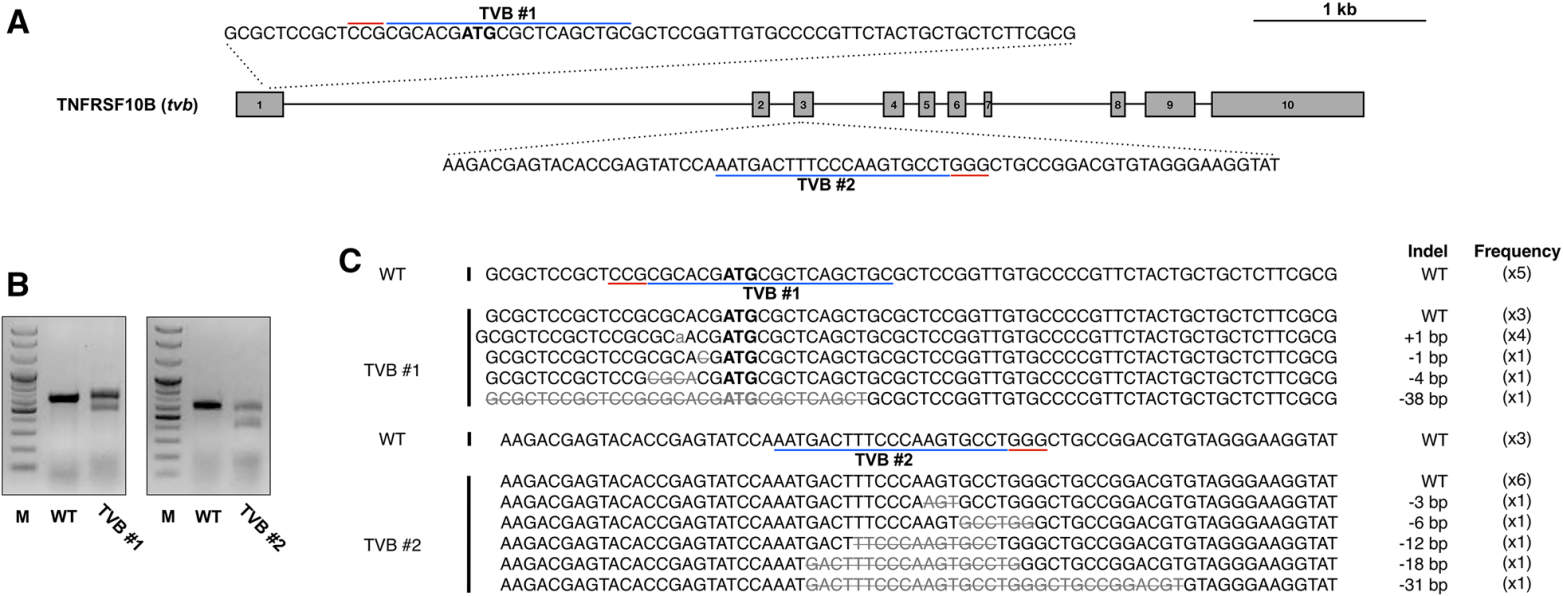
**Genetic modification of** ***tvb*** **by CRISPR/Cas9 in DF-1 cells. A** Gene structure of *tvb* (TNFRSF10B) and recognition sites of TVB#1 and TVB#2 CRISPR/Cas9 vectors. Blue bars indicate guide RNA recognition sites, and red bars indicate protospacer-adjacent motif (PAM) sequences. Scale bar = 1 kb. **B** T7E1 assay for DF-1 cells transfected with TVB#1 and TVB#2 CRISPR/Cas9 vectors. Bands cleaved by T7E1 endonuclease were seen in the experimental groups. **C** Sequencing analysis of transfected DF-1 cells using the TA cloning method. Grey letters indicate insertions, and grey letters with lines indicate deletions. Indel mutations and their frequencies are presented. Blue bars indicate guide RNA recognition sites, and red bars indicate PAM sequences. Wild type (WT) DF-1 cells were used as the control.

To efficiently disrupt *tvb*, we designed two CRISPR/Cas9 vectors targeting two different sites within the gene. The TVB#1 vector (TVB#1) was designed to target the ATG sequence of *tvb*, which can inhibit gene translation. The TVB#2 vector (TVB#2) was designed to target exon 3 of *tvb*, which can cause frame shift mutations resulting in production of a stop codon in CRDs of TVB receptors [7] (Figure 2A). DF-1 cells transfected with TVB#1 and TVB#2 were successfully selected 7 days post-transfection using G418, and T7E1 analysis showed that the transfected cells had indel mutations in targeted loci (Figure 2B). The mutations were analyzed using the TA cloning method, and the mutation efficiencies of the two targeted loci were 70 and 45.5% in DF-1 cells transfected with TVB#1 and TVB#2, respectively (Figure 2C). The patterns of mutations were diverse in both experimental groups. In DF-1 cells transfected with TVB#1, we identified both deletions and insertions. In DF-1 cells transfected with TVB#2, only deletions mutations were identified (Figure 2C).

### Establishment of single DF-1 clones and genomic DNA analysis

To establish single *tvb*-mutated DF-1 clones, we picked single cells from DF-1 cells transfected with TVB#1 and TVB#2, respectively. The picked single cells became attached and actively proliferated (Additional file 1: A). We established several different clones from both TVB#1- and TVB#2- transfected DF-1 cells. Unfortunately, we obtained only clones with a 1 bp insertion before the ATG sequence from DF-1 cells transfected with TVB#1 (Additional file 2); therefore, we evaluated only the clones from DF-1 cells transfected with TVB#2. For 3‒4 weeks, a total of 21 clones from DF-1 cells transfected with TVB#2 were established (Additional file 1: B), and the clones were then sequenced. Sequencing of the PCR products revealed a single clear peak in all samples, indicating that all DF-1 clones had diverse bi-allelic mutation patterns (Table 2; Additional file 3). Specifically, clone #17 had a 44 bp insertion in targeted loci, and clones #13, #16 and #28 had 15, 12 and 15 bp deletions, respectively, which could not induce frame shift mutations. Other clones (#2, #10, #14, #18, #19, #21, #23, #25 and #27) had deletions in targeted loci that caused frame shift mutations (Table 2).

**Table 2:**
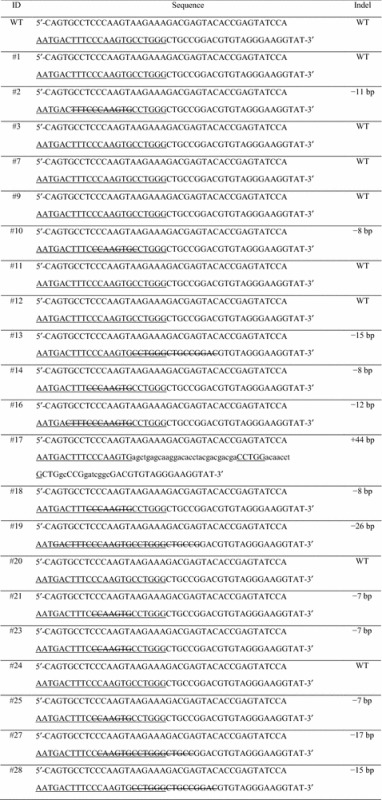
**Sequencing results of** ***tvb*** **-modified DF-1 clones**

Underlining indicates the TVB#2 guide RNA recognition site and protospacer-adjacent motif (PAM) sequences

Strikethrough lines indicate deleted nucleotides and lowercase letters indicate inserted nucleotides

### ALV subgroup B infection in single DF-1 clones and flow cytometry

**Figure 3:**
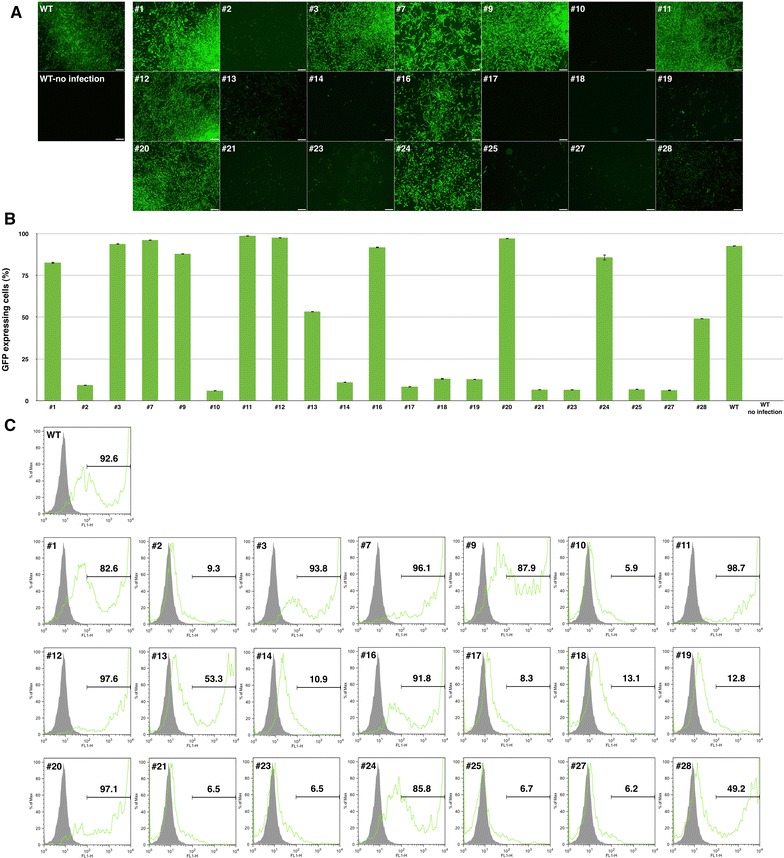
**Viral infection of DF-1 clones and flow cytometric analysis. A** GFP expression in virus-infected DF-1 cells. Twenty-one DF-1 clones were evaluated under a fluorescence microscope. Scale bar = 200 µm. **B**, **C** Flow cytometric analysis of virus-infected DF-1 clones. WT DF-1 cells were used as the control.

To verify the susceptibility of DF-1 clones to ALV subgroup B infection, clones from DF-1 cells transfected with TVB#2 were infected with ALV subgroup B. Strong GFP expression was detected in nine different clones (#1, #3, #7, #9, #11, #12, #16, #20 and #24) compared with WT DF-1 cells at 4 days post-infection. We also found markedly lower levels of GFP expression in clones #2, #10, #14, #17, #18, #19, #21, #23, #25 and #27 and moderate levels of GFP expression in clones #13 and #28 (Figure 3A). The results of flow cytometric analysis showed that the proportions of GFP-expressing cells in DF-1 clones #1, #3, #7, #9, #11, #12, #16, #20 and #24 were 82.6, 93.8, 96.1, 87.9, 98.7, 97.6, 91.8, 97.1 and 85.8%, respectively. The proportions of GFP-expressing cells in clones #2, #10, #14, #17, #18, #19, #21, #23, #25 and #27, which exhibited low levels of GFP expression, were 9.3, 5.9, 10.9, 8.3, 13.1, 12.8, 6.5, 6.5, 6.7 and 6.2%, respectively. The proportions of GFP-expressing cells in clones #13 and #28, which exhibited moderate levels of GFP expression, were 53.3 and 49.2%, respectively (Figures 3B and C).

### Amino acid sequence analysis

**Figure 4:**
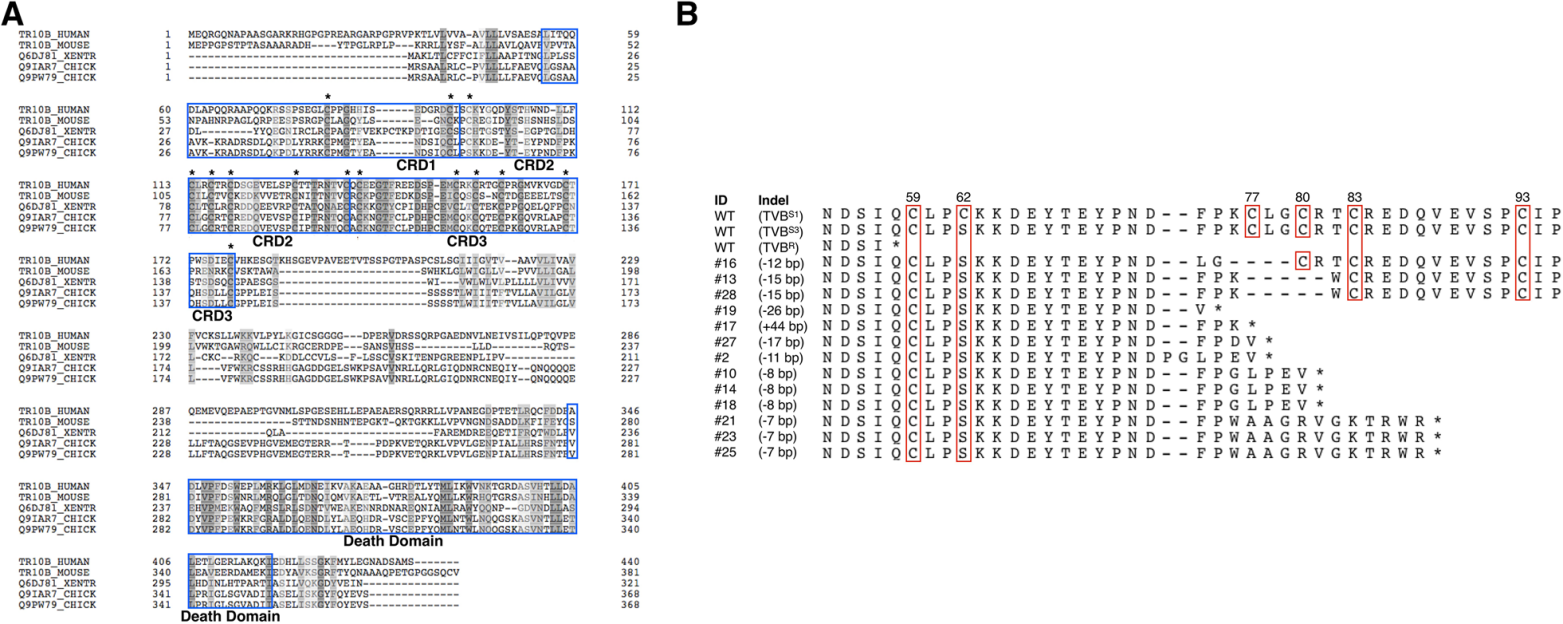
**Analysis of deduced amino acid sequences of** ***tvb*** **-modified DF-1 clones. A** Sequence alignment of human DR5 TRAIL receptor (NP_003833.4), mouse Tnfrsf10b (NP_064671.2), western clawed frog tnfrsf10b (NP_001004894.1) and chicken TVB^S1^ (NP_989446.2). Blue boxes indicate cysteine-rich domains (CRDs) and death domains (DDs); shaded boxes indicated similarities among amino acid sequences. Asterisks indicate conserved cysteine residues. **B** Sequence alignment of deduced amino acids in *tvb*-modified DF-1 clones. Red boxes indicate conserved cysteine residues, and numbers indicate the order of the amino acids in TVB proteins. Asterisks indicate stop codons. WT with *tvb* ^*s1*^, *tvb* ^*s3*^ and *tvb* ^*r*^ genotypes were used as the control.

To identify the reason for the significantly reduced ALV group B susceptibility in *tvb*-mutated DF-1 clones, we analyzed their amino acid sequences. First, we compared the sequence of tumor necrosis factor receptor superfamily member 10B (TNFRSF10B) among humans, mice, frogs and chickens (TVB^S1^ and TVB^S3^). Sequence alignment analysis revealed highly conserved amino acid sequences in CRDs located in the extracellular receptor domain and cytoplasmic death domain (DD), which mediates the apoptosis signaling pathway. In particular, we located highly conserved cysteine residues in CRDs that formed bisulfide bonds (Figure 4A).

Next, we deduced the amino acid sequences of *tvb*-mutated DF-1 clones. Amino acid sequence analysis showed that clones #10, #14 and #18 shared the same amino acid sequences, as did clones #21, #23 and #25. Clones had mutations that generated an in-frame stop codon resulting in the production of a truncated protein similar to that of the WT *tvb* ^*r*^. Clones #13 and #28, which expressed moderate levels of GFP, possessed the same mutations, including five amino acid deletions and one amino acid substitution from the 77^th^ to 82^nd^ amino acid positions of the TVB receptor. Clone #16, which expressed high levels of GFP, had four amino acids deletions from the 76th to 79th amino acid positions of the TVB receptor (Figure 4B). The mutations of nucleotides and deduced amino acids, and results of virus challenge in *tvb*-modified DF-1 clones were summarized in Table 3.

**Table 3:** **Mutations of nucleotides and deduced amino acids, and results of virus challenge in** ***tvb*** **-modified DF-1 clones**

ID	Indel	Amino acid mutation	GFP expression (%)
WT	WT	No mutation	Strong (92.6)
#16	−12 bp	4 amino acid deletion (76th to 79th positions)	Strong (91.8)
#13	−15 bp	5 amino acid deletion (77th to 80th positions)	Moderate (53.3)
#28	−15 bp	5 amino acid deletion (77th to 80th positions)	Moderate (49.2)
#19	−26 bp	Premature stop codon	Low (12.8)
#17	+44 bp	Premature stop codon	Low (8.3)
#27	−17 bp	Premature stop codon	Low (6.2)
#2	−11 bp	Premature stop codon	Low (9.3)
#10	−8 bp	Premature stop codon	Low (5.9)
#14	−8 bp	Premature stop codon	Low (10.9)
#18	−8 bp	Premature stop codon	Low (13.1)
#21	−7 bp	Premature stop codon	Low (6.5)
#23	−7 bp	Premature stop codon	Low (6.5)
#25	−7 bp	Premature stop codon	Low (6.7)

## Discussion

The acquisition of complete disease resistance is the ultimate goal of the agricultural industry and human society. However, despite the development of vaccines and improvements in quarantine facilities, complete disease control is elusive owing to economic costs and the rapid evolution of viruses. To overcome these limitations, targeted DNA modification using CRISPR/Cas9 technology has been used against a variety of viruses [22–27]. This system specifically targets and modifies the structure of viral genomes or host viral receptor genes and may therefore be used for efficient viral disease prevention without off-target events [28]. Therefore, in the present study, we used the CRISPR/Cas9-mediated genome editing method to confer resistance to ALV subgroup B in DF-1 chicken fibroblasts.

To evaluate the feasibility of CRISPR/Cas9-mediated genome editing for engineering resistance to ALV subgroup B, we first designed two CRISPR/Cas9 vectors specifically targeting two *tvb* loci for genetic mutations (Figure 2A). Chickens that have a naturally occurring single bp mutation in *tvb* are reported to be resistant to infection by ALV subgroups B, D and E, and this is thought to be because of the creation of a premature stop codon in the CRD1 domain [7]. To artificially generate a premature stop codon in *tvb* in DF-1 cells, we targeted the 3’ region of the CRD1 coding region. We also targeted the *tvb* start codon to achieve total disruption of *tvb* receptor protein production. We found that targeted loci were successfully modified to possess indel mutations, and most mutations occurred in regions neighboring protospacer-adjacent motif sequences, which is consistent with previous research (Figure 2C) [17].

Next, we cultured DF-1 clones harboring *tvb* mutations to obtain clones with homozygous genotypes (Additional file 1). To avoid misinterpretation due to mixed genotypes during the virus challenge experiment, we verified DF-1 genotypes by sequencing analysis of PCR products. In total, 21 single DF-1 clones from TVB#2-transfected DF-1 cells were established. Of these, 12 had indel mutations in *tvb*, of which 11 were deletions, and only 1 was an insertion (Table 2). As previous research has reported, our results showed that the deletions were mostly CRISPR/Cas9-mediated mutations [29]. Sequencing analysis showed that all DF-1 clones had bi-allelic mutations, which was also consistent with previous research (Additional files 2 and 3) [30, 31]. However, using TVB#1 we were able to obtain only DF-1 clones that had 1 bp insertions in the 5’ region (see Additional file 2). The sequencing results for TVA#1-transfected DF-1 cells suggested that more clones were required for analysis or that precise genome editing mediated by homologous recombination was required to obtain clones with mutations in ATG sequences.

To evaluate the viral susceptibility of established single DF-1 cells, the cells were infected with ALV subgroup B produced by replication-competent ALV long terminal repeat (LTR) using a splice acceptor (RCAS) vector. The RCAS vector used in the present study is replication-competent in avian cells, and the vector spreads rapidly and infects most cells in vitro within a short period of time [32]. Exploiting these characteristics, we successfully produced ALV subgroup B in DF-1 cells (Figure 1B). In the virus challenge experiments, single DF-1 clones with mutations (#2, #10, #13, #14, #17, #18, #19, #21, #22, #23, #25, #27 and #28) had significantly lower levels of GFP expression compared with WT DF-1 cells. This suggests that genetic modification of *tvb* affected ALV subgroup B susceptibility in DF-1 cells. However, in this paper, we could not get the absolute resistant against to ALV subgroup B. 5.9% (#10) was the least expression of GFP among the clones (Figure 3C). The results may come from different between genotype of chicken that has *tvb* ^*r*^ and those of *tvb*-modified DF-1 clones. Analysis of deduced amino acid shows *tvb*-modified DF-1 clones have still 59th cysteine residue that is important in ALV subgroup B entry [7]. Precise modification of the DF-1 *tvb* gene to *tvb* ^*r*^ genotypes may cause absolute resistant to the virus. Furthermore, the results can come from difference between in vivo and in vitro system. Previous report revealed that the chickens that have resistant to ALV subgroup A do not have any proliferation of sarcomas even after 42 dpi even 40% of their CEF express GFP at 7 dpi by ALV subgroup A infection [6]. The results suggest that there is difference between in vivo and in vitro validation. To identify resistance to ALV subgroup B, genome-edited chicken needs to be produced and validated comparing with the chickens that have *tvb* ^*r*^ genotype.

Analysis of deduced amino acid sequences revealed that genetic modifications of *tvb* generated a premature stop codon in the CRD2 domain (Figure 4B). This suggested that the artificially generated stop codon plays a crucial role in ALV subgroup B entry into host cells, similar to virus-resistant WL CEFs [7]. Resistance to human immunodeficiency virus (HIV) infection is also associated with mutations in a host receptor, CCR5. Individuals who have a 32 bp deletion that creates a premature stop codon in the CCR5 receptor are resistant to HIV [33, 34]. Therefore, our results support the notion that amino acid substitutions, particularly those that generate premature stop codons in host receptors, can abolish the functions of these receptors in viral interactions.

Interestingly, DF-1 clone #16 contained 91.8% GFP-expressing cells, although *tvb* gene contained a 12 bp deletion. This suggests that the deletion of four amino acids from the 76^th^ to 79^th^ positions of the TVB receptor does not significantly alter viral susceptibility. Comparing this clone with clones #13 and #28, which possessed a 15 bp deletion, suggested that cysteine 80 and arginine 81 within the TVB receptor play an important role in ALV subgroup B entry into host cells. Indeed, cysteine residues in CRDs are crucial for viral entry in several organisms. For example, the attachment of herpes simplex virus (HSV), equine infectious anemia virus (EIAV), feline immunodeficiency virus (FIV) and rabies virus (RABV) to host cells is mediated by the CRDs of their specific TNF receptors (herpes virus entry mediator [TANFRSF14] for HSV and EIAV, Ox40 [CD134, TNFRSF4] for FIV and NTRp75 for RABV). Cysteine residues in these CRDs are highly conserved, and mutations in these residues cause conformational changes in the extracellular regions of the receptors, altering their affinity to ligands [35]. In avian species, a cysteine-to-tryptophan substitution in the low-density lipoprotein receptor-like region of TVA drastically reduces the binding affinity of ALV subgroup A; similarly, a cysteine-to-serine mutation at position 62 in the TVB^S3^ receptor reduces susceptibility to ALV subgroup E [4, 36]. Collectively, the results of the present study and previous research suggest the importance of bisulfide bonds in CRDs mediated by cysteine residues. However, studies investigating the precise replacement of cysteine 80 by homologous recombination are required to provide further support for our hypothesis.

## Conclusion

In the present study, we demonstrated the feasibility of CRISPR/Cas9-mediated genome modification for engineering resistance to ALV subgroup B. We efficiently modified DF-1 chicken fibroblasts using the CRISPR/Cas9 system and confirmed that modified DF-1 cells acquired resistance to ALV subgroup B. These results indicate that generating premature stop codons in the CRDs of TVB receptors can alter viral susceptibility, and that cysteine residues forming bisulfide bonds in CRDs may play important roles in determining susceptibility to ALV subgroup B. Furthermore, our results show that the CRISPR/Cas9 system can be used to efficiently modify the avian genome and establish novel avian cell lines, including virus-resistant chicken cell lines, mediated by primordial germ cells with germline competency. Furthermore, we expect that this system will facilitate the study of virus-host interactions not only in avian species but also in humans, for example, HIV and its relationship with the human CCR5 co-receptor [33, 34].

## Additional files

**Additional file 1.** **Establishment of** ***tvb*** **-modified DF-1 clones.** (A) DF-1 cell morphology during *in vitro* culture. Scale bar = 50 µm. (B) Establishment of 21 individual DF-1 clones. Wild type (WT) DF-1 cells were used as the control. Scale bar = 200 µm.

**Additional file 2.** **Sequencing results of TVB#1-transfected DF-1 clones with chromatography.** Wild type (WT) DF-1 cells with *tvb* ^*s3*^ genotypes were used as the control. The red arrow indicates the guide RNA recognition site, and red rectangles indicate insertions in *tvb*.

**Additional file 3.** **Sequencing results of TVB#2-transfected DF-1 clones using chromatography.** Wild type (WT) DF-1 cells with *tvb* ^*s3*^ genotypes were used as the control. The red arrow indicates the guide RNA recognition site, and orange rectangles indicate specific single nucleotide polymorphisms in *tvb*.

## Abbreviations

CRDs: cysteine rich domains
ALV: avian leukosis virus
*tvb*: tumor virus locus B
*tva*: tumor virus locus A
*tvc*: tumor virus locus C
W38: tryptophan 38
CRISPR/Cas9: clustered regularly interspaced short palindromic repeats (CRISPR)/CRISPR-associated
DMEM: Dulbecco’s minimum essential medium
FBS: fetal bovine serum
ABAM: antibiotic–antimycotic
WL: White Leghorn
CEF: chicken embryonic fibroblast
EDTA: ethylenediaminetetraacetic acid
dpi: days post-infection
SAS: the statistical analysis system
ANOVA: analysis of variance
WT: wild type
GFP: green fluorescent protein
TNFRSF10B: tumor necrosis factor receptor superfamily member 10B
DD: death domain
PAM: protospacer adjacent motif
LTR: long terminal repeat
RCAS: replication-competent ALV LTR with a splice acceptor
HIV: human immunodeficiency virus
EIAV: equine infectious anemia virus
FIV: feline immunodeficiency virus
RABS: rabies virus
HVEM: herpes virus entry mediator
LDLR: low-density lipoprotein receptor
PGC: primordial germ cell
gRNA: guide RNA

## References

[1] Justice J, Beemon KL (2013). Avian retroviral replication. Curr Opin Virol.

[2] Payne LN, Howes K, Gillespie AM, Smith LM (1992). Host range of *Rous sarcoma* virus pseudotype RSV(HPRS-103) in 12 avian species: support for a new avian retrovirus envelope subgroup, designated J. J General Virol.

[3] Payne LN, Nair V (2012). The long view: 40 years of avian leukosis research. Avian Pathol.

[4] Elleder D, Melder DC, Trejbalova K, Svoboda J, Federspiel MJ (2004). Two different molecular defects in the Tva receptor gene explain the resistance of two tvar lines of chickens to infection by subgroup A avian sarcoma and leukosis viruses. J Virol.

[5] Chen W, Liu Y, Li H, Chang S, Shu D, Zhang H, Chen F, Xie Q (2015). Intronic deletions of tva receptor gene decrease the susceptibility to infection by avian sarcoma and leukosis virus subgroup A. Sci Rep.

[6] Reinišová M, Plachý J, Trejbalová K, Šenigl F, Kučerová D, Geryk J, Svoboda J, Hejnar J (2012). Intronic deletions that disrupt mRNA splicing of the tva receptor gene result in decreased susceptibility to infection by avian sarcoma and leukosis virus subgroup A. J Virol.

[7] Klucking S, Adkins HB, Young JA (2002). Resistance to infection by subgroups B, D, and E avian sarcoma and leukosis viruses is explained by a premature stop codon within a resistance allele of the tvb receptor gene. J Virol.

[8] Reinisová M, Senigl F, Yin X, Plachy J, Geryk J, Elleder D, Svoboda J, Federspiel MJ, Hejnar J (2008). A single-amino-acid substitution in the TvbS1 receptor results in decreased susceptibility to infection by avian sarcoma and leukosis virus subgroups B and D and resistance to infection by subgroup E in vitro and in vivo. J Virol.

[9] Elleder D, Stepanets V, Melder DC, Senigl F, Geryk J, Pajer P, Plachy J, Hejnar J, Svoboda J, Federspiel MJ (2005). The receptor for the subgroup C avian sarcoma and leukosis viruses, Tvc, is related to mammalian butyrophilins, members of the immunoglobulin superfamily. J Virol.

[10] Chai N, Bates P (2006). Na+/H+ exchanger type 1 is a receptor for pathogenic subgroup J avian leukosis virus. Proc Natl Acad Sci U S A.

[11] Kucerová D, Plachy J, Reinisová M, Senigl F, Trejbalová K, Geryk J, Hejnar J (2013). Nonconserved tryptophan 38 of the cell surface receptor for subgroup J avian leukosis virus discriminates sensitive from resistant avian species. J Virol.

[12] Jinek M, Chylinski K, Fonfara I, Hauer M, Doudna JA, Charpentier E (2012). A programmable dual-RNA—guided DNA endonuclease in adaptive bacterial immunity. Science.

[13] Hwang WY, Fu Y, Reyon D, Maeder ML, Tsai SQ, Sander JD, Peterson RT, Yeh JR, Joung JK (2013). Efficient genome editing in zebrafish using a CRISPR-Cas system. Nat Biotechnol.

[14] Tan W, Carlson DF, Lancto CA, Garbe JR, Webster DA, Hackett PB, Fahrenkrug SC (2013). Efficient nonmeiotic allele introgression in livestock using custom endonucleases. Proc Natl Acad Sci U S A.

[15] Wang H, Yang H, Shivalila CS, Dawlaty MM, Cheng AW, Zhang F, Jaenisch R (2013). One-step generation of mice carrying mutations in multiple genes by CRISPR/Cas-mediated genome engineering. Cell.

[16] Gao Y, Wu H, Wang Y, Liu X, Chen L, Li Q, Cui C, Liu X, Zhang J, Zhang Y (2017). Single Cas9 nickase induced generation of NRAMP1 knockin cattle with reduced off-target effects. Genome Biol.

[17] Oishi I, Yoshii K, Miyahara D, Kagami H, Tagami T (2016). Targeted mutagenesis in chicken using CRISPR/Cas9 system. Sci Rep.

[18] Dimitrov L, Pedersen D, Ching KH, Yi H, Collarini EJ, Izquierdo S, van de Lavoir MC, Leighton PA (2016). Germline gene editing in chickens by efficient CRISPR-mediated homologous recombination in primordial germ cells. PLoS ONE.

[19] Sakuma T, Nishikawa A, Kume S, Chayama K, Yamamoto T (2014). Multiplex genome engineering in human cells using all-in-one CRISPR/Cas9 vector system. Sci Rep.

[20] Lee HJ, Lee HC, Kim YM, Hwang YS, Park YH, Park TS, Han JY (2016). Site-specific recombination in the chicken genome using Flipase recombinase–mediated cassette exchange. FASEB J.

[21] Park TS, Lee HJ, Kim KH, Kim JS, Han JY (2014). Targeted gene knockout in chickens mediated by TALENs. Proc Natl Acad Sci U S A.

[22] Ali Z, Abulfaraj A, Idris A, Ali S, Tashkandi M, Mahfouz MM (2015). CRISPR/Cas9-mediated viral interference in plants. Genome Biol.

[23] Hu W, Kaminski R, Yang F, Zhang Y, Cosentino L, Li F, Luo B, Alvarez-Carbonell D, Garcia-Mesa Y, Karn J, Mo X, Khalili K (2014). RNA-directed gene editing specifically eradicates latent and prevents new HIV-1 infection. Proc Natl Acad Sci USA.

[24] Price AA, Sampson TR, Ratner HK, Grakoui A, Weiss DS (2015). Cas9-mediated targeting of viral RNA in eukaryotic cells. Proc Natl Acad Sci USA.

[25] Suenaga T, Kohyama M, Hirayasu K, Arase H (2014). Engineering large viral DNA genomes using the CRISPR-Cas9 system. Microbiol Immunol.

[26] Zhen S, Hua L, Liu YH, Gao LC, Fu J, Wan DY, Dong LH, Song HF, Gao X (2015). Harnessing the clustered regularly interspaced short palindromic repeat (CRISPR)/CRISPR-associated Cas9 system to disrupt the hepatitis B virus. Gene Ther.

[27] Burkard C, Lillico SG, Reid E, Jackson B, Mileham AJ, Ait-Ali T, Whitelaw CB, Archibald AL (2017). Precision engineering for PRRSV resistance in pigs: macrophages from genome edited pigs lacking CD163 SRCR5 domain are fully resistant to both PRRSV genotypes while maintaining biological function. PLoS Pathog.

[28] Khalili K, White MK, Jacobson JM (2017). Novel AIDS therapies based on gene editing. Cell Mol Life Sci.

[29] Varshney GK, Pei W, LaFave MC, Idol J, Xu L, Gallardo V, Carrington B, Bishop K, Jones M, Li M, Harper U, Huang SC, Prakash A, Chen W, Sood R, Ledin J, Burgess SM (2015). High-throughput gene targeting and phenotyping in zebrafish using CRISPR/Cas9. Genome Res.

[30] Jao LE, Wente SR, Chen W (2013). Efficient multiplex biallelic zebrafish genome editing using a CRISPR nuclease system. Proc Natl Acad Sci USA.

[31] Wang X, Zhou J, Cao C, Huang J, Hai T, Wang Y, Zheng Q, Zhang H, Qin G, Miao X, Wang H, Cao S, Zhou Q, Zhao J (2015). Efficient CRISPR/Cas9-mediated biallelic gene disruption and site-specific knockin after rapid selection of highly active sgRNAs in pigs. Sci Rep.

[32] Hughes SH (2004). The RCAS vector system. Folia Biol.

[33] Liu R, Paxton WA, Choe S, Ceradini D, Martin SR, Horuk R, MacDonald ME, Stuhlmann H, Koup RA, Landau NR (1996). Homozygous defect in HIV-1 coreceptor accounts for resistance of some multiply-exposed individuals to HIV-1 infection. Cell.

[34] Samson M, Libert F, Doranz BJ, Rucker J, Liesnard C, Farber CM, Saragosti S, Lapoumeroulie C, Cognaux J, Forceille C, Muyldermans G, Verhofstede C, Burtonboy G, Georges M, Imai T, Rana S, Yi Y, Smyth RJ, Collman RG, Doms RW, Vassart G, Parmentier M (1996). Resistance to HIV-1 infection in caucasian individuals bearing mutant alleles of the CCR-5 chemokine receptor gene. Nature.

[35] Kinkade A, Ware CF (2006). The DARC conspiracy—virus invasion tactics. Trends Immunol.

[36] Adkins HB, Blacklow SC, Young JA (2001). Two functionally distinct forms of a retroviral receptor explain the nonreciprocal receptor interference among subgroups B, D, and E avian leukosis viruses. J Virol.

