# Supplementary figures and images for: Acquisition of resistance to avian leukosis virus subgroup B through mutations on tvb cysteine-rich domains in DF-1 chicken fibroblasts
Source: Vet Res. 2017 Sep 13;48:48. doi: 10.1186/s13567-017-0454-1 (PMC5598054; doi:10.1186/s13567-017-0454-1)

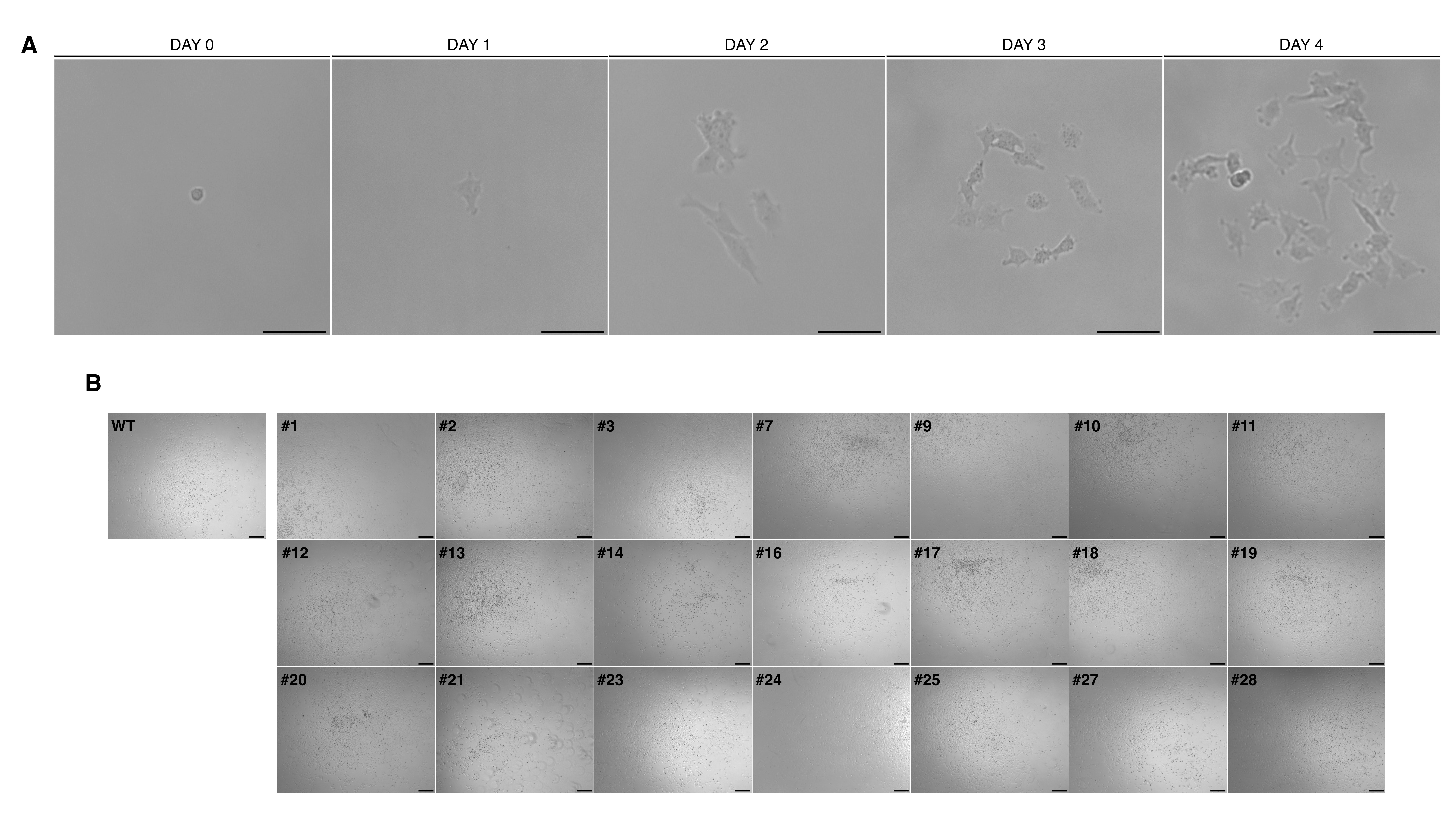

Supplement: Supplementary file 1 — Additional file 1. Establishment of tvb -modified DF-1 clones. (A) DF-1 cell morphology during in vitro culture. Scale bar = 50 µm. (B) Establishment of 21 individual DF-1 clones. Wild type (WT) DF-1 cells were used as the control. Scale bar = 200 µm. [file 13567_2017_454_MOESM1_ESM.jpg]

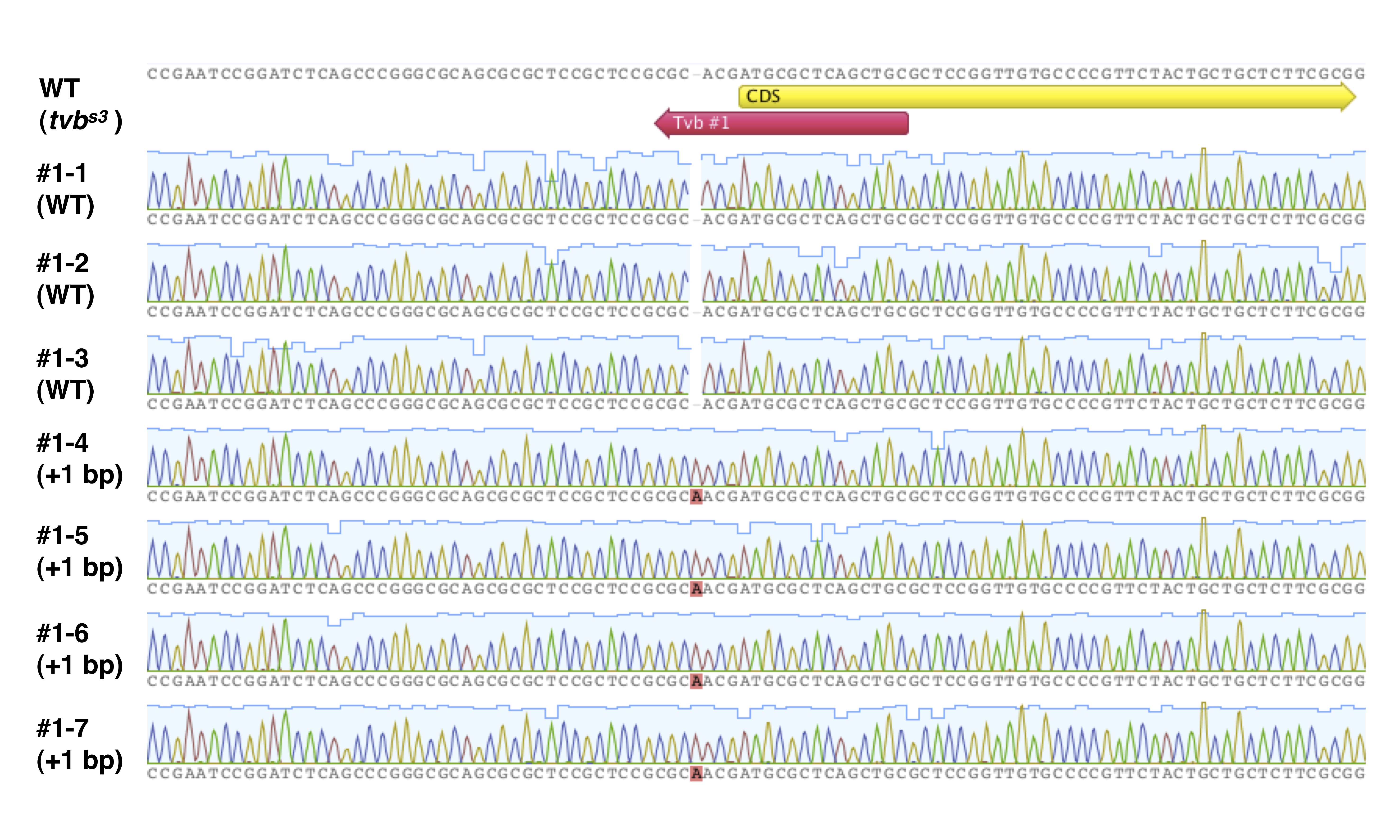

Supplement: Supplementary file 2 — Additional file 2. Sequencing results of TVB#1-transfected DF-1 clones with chromatography. Wild type (WT) DF-1 cells with tvb s3 genotypes were used as the control. The red arrow indicates the guide RNA recognition site, and red rectangles indicate insertions in tvb. [file 13567_2017_454_MOESM2_ESM.jpg]

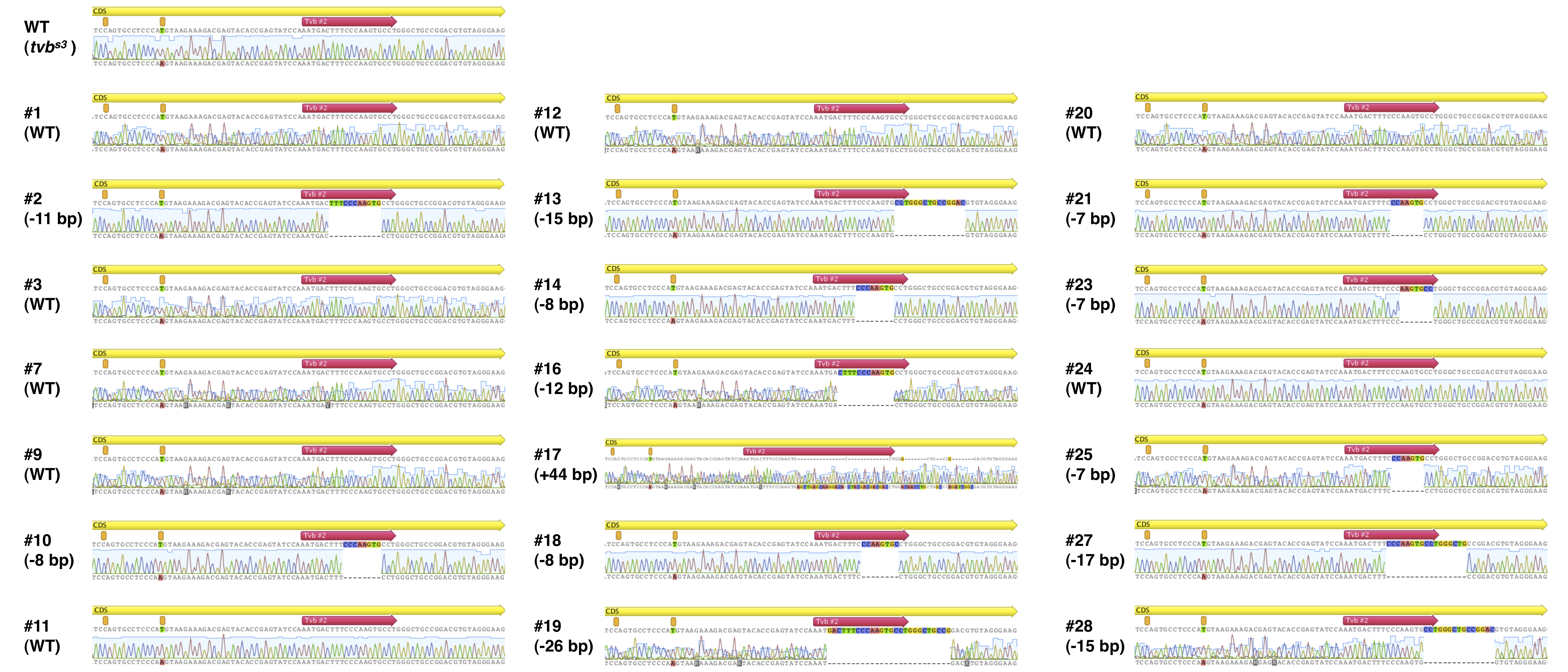

Supplement: Supplementary file 3 — Additional file 3. Sequencing results of TVB#2-transfected DF-1 clones using chromatography. Wild type (WT) DF-1 cells with tvb s3 genotypes were used as the control. The red arrow indicates the guide RNA recognition site, and orange rectangles indicate specific single nucleotide polymorphisms in tvb. [file 13567_2017_454_MOESM3_ESM.jpg]
